# A Novel Technique for Prealignment in Multimodality Medical Image Registration

**DOI:** 10.1155/2014/726852

**Published:** 2014-05-22

**Authors:** Wu Zhou, Lijuan Zhang, Yaoqin Xie, Changhong Liang

**Affiliations:** ^1^Shenzhen Key Laboratory for Low-Cost Healthcare, Shenzhen Institutes of Advanced Technology, Chinese Academy of Sciences, Shenzhen 518055, China; ^2^Department of Radiology, Guangdong General Hospital, Guangzhou 510080, China

## Abstract

Image pair is often aligned initially based on a rigid or affine transformation before a deformable registration method is applied in medical image registration. Inappropriate initial registration may compromise the registration speed or impede the convergence of the optimization algorithm. In this work, a novel technique was proposed for prealignment in both monomodality and multimodality image registration based on statistical correlation of gradient information. A simple and robust algorithm was proposed to determine the rotational differences between two images based on orientation histogram matching accumulated from local orientation of each pixel without any feature extraction. Experimental results showed that it was effective to acquire the orientation angle between two unregistered images with advantages over the existed method based on edge-map in multimodalities. Applying the orientation detection into the registration of CT/MR, T1/T2 MRI, and monomadality images with respect to rigid and nonrigid deformation improved the chances of finding the global optimization of the registration and reduced the search space of optimization.

## 1. Introduction

Image registration is the spatial mapping of corresponding locations between different images with broad applications in neurosurgery and radiotherapy [1]. Medical image registration techniques have undergone continuous development and extensive research over decades [2–7]. In general applications of medical image registration, an image pair is often aligned initially based on a rigid or affine transformation before a nonrigid warp is applied. The initial rigid transformation is performed to fast approximate the global deformation between the images, and the subsequent nonrigid warp is supposed to refine the local deformation, such as free-form deformation (FFD) [8] or Demons [9, 10]. An initial preregistration may be defined manually or automatically [11, 12]. In the image moments-based method, the rotational difference is determined by the second-order central moment, and the translation is determined by the displacement of the centroid of corresponding objects in two images. Manual initialization by users may be accurate but impractical for clinicians. The application of image moments for initial estimation needs presegmentation of objects for moment calculation and often complicates the registration. In particular, the accurate segmentation of corresponding structures in Multimodality images may be impossible to complete. Feature-based registration methods [13, 14] are prone to fail due to the less availability of the corresponding features between multimodality images.

It was previously reported that orientation difference estimation based on edge information [15] with Canny filter depends on the similarity between the two images. Automatic multimodal image registration remains challenging in terms of consistency in intensity or contrast patterns and the existence of nonoverlapping regions between images. In addition, the method was limited for rigid transformation due to the utilization of polar and Fourier transformations in frequency domain, thus not much robust to partial occlusion and complex global or local deformation between images. Since the unregistered images may contain considerable global and local nonlinear deformations in addition to global rigid deformation, orientation detection between images has to be robust to considerable complex deformation, partial occlusion, and illumination changes.

In this study, the feasibility of calculating the orientation difference between two unregistered images is explored to facilitate the image registration without any feature extraction. The challenges lie in the lack of abundant corresponding characteristics between multimodality images. Tissue boundaries may vary in corresponding multimodality images, but the distribution of gradient information often has considerable similarities. The orientation difference between multimodality images is expected to be determined by the orientation histogram of gradient information. Gradient information has been widely used in the field of image registration [16–21]. Histogram of gradient (HOG) has been widely used in computer vision and pattern recognition [22], for example, feature point descriptors [23] and hand gesture recognition [24]. The applications of HOG in the previous work are mainly designed for local descriptions in images. The orientation bins in the histogram are often 4 or 8 to describe the local content of nearest neighborhood around the feature point.

Contrast to all previously proposed methods based on gradient information, gradient information is adopted to estimate the global orientation difference between images in this work. The estimation of global rotational difference is casted as the problem of gradient magnitude weighted orientation histogram matching. A robust technique for histogram matching based on L1 norm and L2 norm is also provided, which is robust to local deformation, partial occlusion, and illumination change in addition to noise in images. Normalizing the gradient magnitude weighted orientation histogram allows estimating the orientation difference across scale differences of images. The main contribution of the work lies in the fact that the global orientation is estimated based on the accumulation of local gradient information, and the global orientation difference is formulated as the problem of robust matching of gradient magnitude weighted orientation histograms.

## 2. Material and Methodology

**Figure 1:**
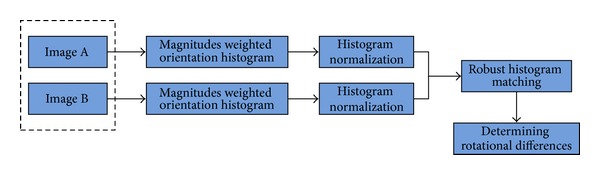
Flowchart of the estimation of the rotational difference.

**Figure 2:**
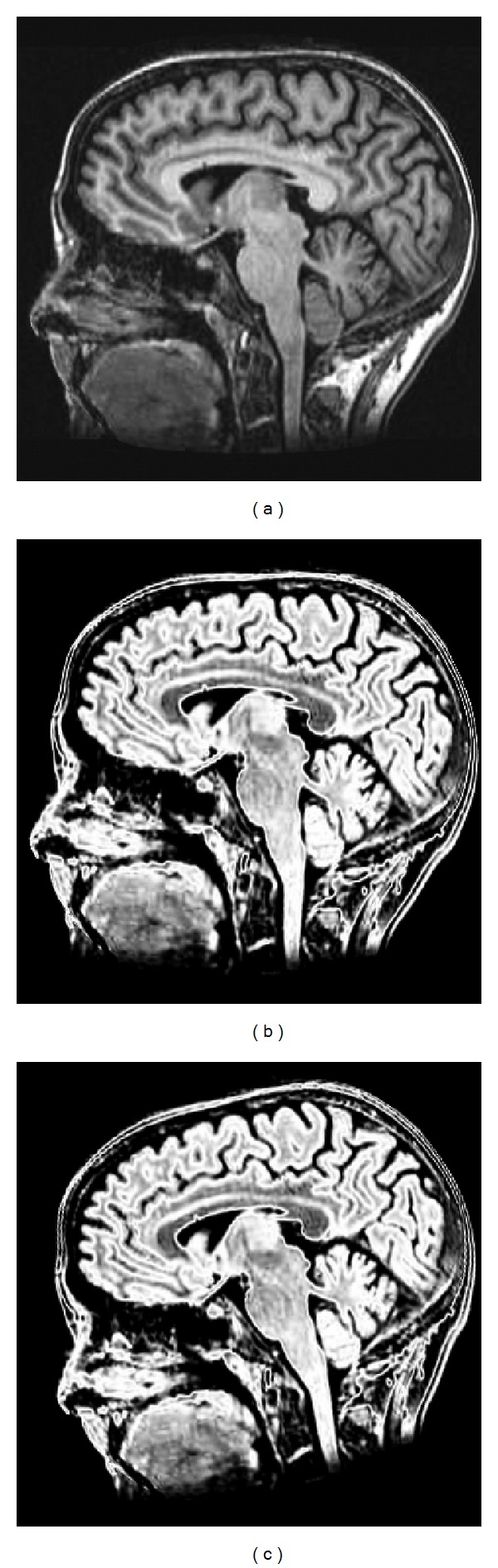
Test MR Brain images. (a) T1 MRI. (b) T2 MRI. (c) Rotate T2 MRI by 11.46°.

The prealignment for multimodality images mainly consists of two components: (1) estimating the rotation and (2) estimating the translation.  Figure 1 shows a schematic of the method for estimating the rotational difference. Initially the gradient magnitudes weighted orientation histograms were constructed for two unregistered images, image A and image B, respectively. Subsequently, the two histograms were normalized for further histogram matching to eliminate the influence of scale differences between images. The process of robust histogram matching was then performed to determine the rotational difference between the images. T1 (T1WI) and T2 (T2WI) weighted magnetic resonance images (MRI) of human brain images were shown in Figure 2 to demonstrate the detailed performance of the proposed algorithm.

### 2.1. The Local Gradient

The local orientation in the image is obtained by calculating the first derivatives in two orthogonal directions and the orientation is determined using the gradient expression illustrated as [25]

(1) $$\begin{array}{c} \nabla f=\frac{\partial f}{\partial x}\hat{x}+\frac{\partial f}{\partial y}\hat{y,} \end{array}$$

where ∂*f*/∂*x* is the gradient in the *x* direction and ∂*f*/∂*y* is the gradient in the *y* direction. The gradient magnitude is calculated by the following formula:

(2) $$\begin{array}{c} m=\sqrt{{\left(\frac{\partial f}{\partial x}\right)}^{2}+{\left(\frac{\partial f}{\partial y}\right)}^{2}.} \end{array}$$

The gradient direction is calculated as

(3) $$\begin{array}{c} \theta =\arctan\left(\frac{\partial f}{\partial x},\frac{\partial f}{\partial y}\right). \end{array}$$

The gradient is computed pixelwise and this operation is carried out by filtering the image with operators such as Sobel in the *x* and *y* directions. To reduce the effect of noisy directions, the partial derivatives in the image are calculated by filtering the image in the *x* and *y* direction with the filters that implement the derivatives of the Gaussian functions. This is achieved by filtering the image with 1D operators that are computed using the expression by the following formula:

(4) $$\begin{array}{c} G\left(x\right)=\frac{1}{\sqrt{2\pi }\sigma }e^{-x^{2}/2\sigma^{2}},\;\;\frac{\partial G}{\partial x}=-\frac{x}{\sigma^{2}}G\left(x\right), \end{array}$$

where *σ* is the standard deviation of the Gaussian function. After calculating the partial derivatives, the weak edges response are eliminated by applying a nonmaximal suppression procedure and the orientation at each pixel is determined by the expression in (3).

**Figure 3:**
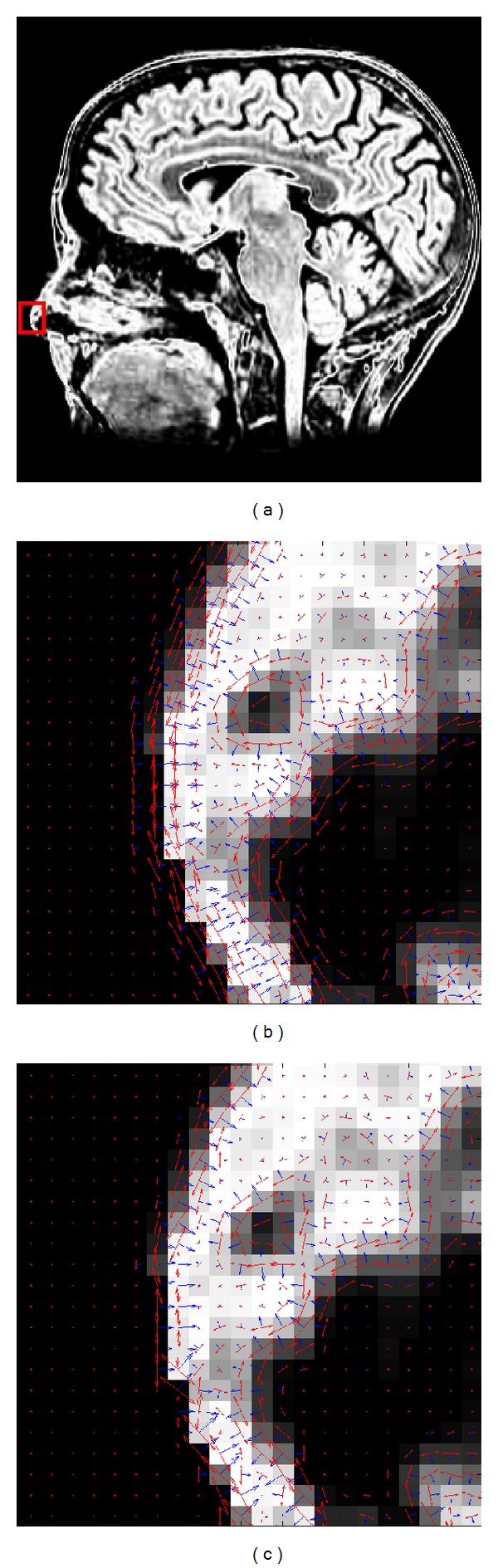
Local gradient calculated by the Gaussian derivative and gradient derivative, respectively. (a) Original image and its portion shown by the red rectangle. (b) Local gradient by Gaussian derivative. (c) Local gradient by gradient derivative. Note that the blue axis shows the local gradient orientation at each pixel, and the length of the axis denotes the value of local gradient magnitude.

One portion (the red rectangle in Figure 3(a)) cropped from T2 MRI image in Figure 2(b) was selected for gradient calculation. Gaussian derivative appeared superior to the gradient derivative (Figure 3). The gradient orientations acquired by Gaussian derivative in the neighborhood area were coherent and insensitive to local noise or intensity changes. In addition, the gradient orientation by Gaussian derivative was reliable and accurate as the derivative calculation was done in continuous rather than a discrete domain.

### 2.2. Magnitudes Weighted Orientation Histogram and Normalization

In this work, the gradient magnitudes weighted orientation histogram was devised to describe the global orientation characteristic of images. The weighted orientation histogram is created with the weight of the gradient direction at a pixel being the gradient magnitude at the pixel. The orientation resolution is set to 1° to balance the accuracy and the robustness of orientation histogram. In addition, the process of histogram normalization eliminates the influence of slightly scale difference or partial occlusion between images.

**Figure 4:**
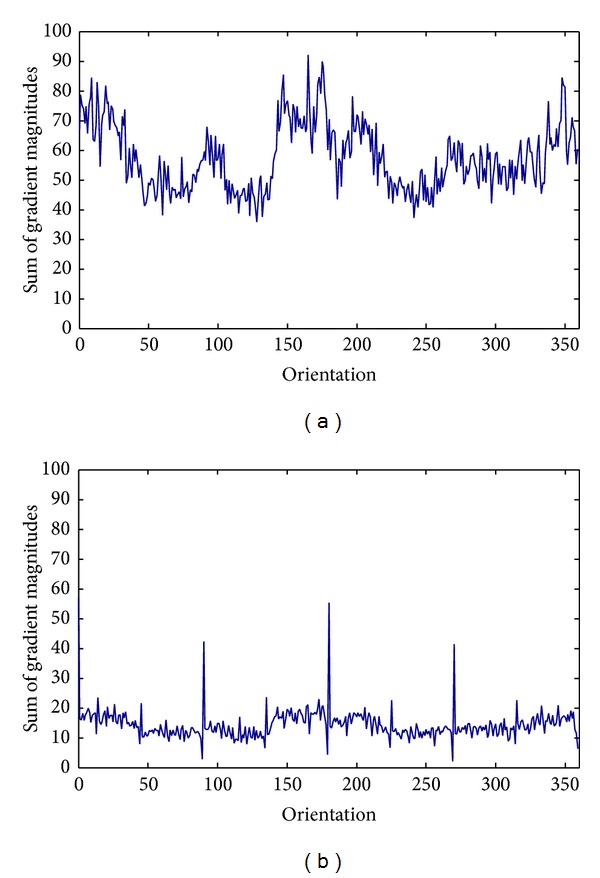
Magnitudes weighted orientation histograms accumulated from local gradients calculated by Gaussian derivative and gradient derivative, respectively. (a) Local gradients calculated by Gaussian derivative. (b) Local gradients calculated by gradient derivative.

The magnitudes weighted orientation histograms accumulated from local gradients calculated by Gaussian derivative and gradient derivative were experimentally compared. In Figure 4, two magnitudes weighted orientation histograms were generated for Brain T2 MR. The magnitudes weighted orientation histogram by Gaussian derivatives distributed more uniformly. However, the Magnitudes weighted orientation histogram by gradient derivative generated large peaks corresponding to 0°, 90°, 180^°,^ and 270°. Comparatively, Gaussian derivative was able to describe the local gradient information, which represented the desired characteristics of local areas.

**Figure 5:**
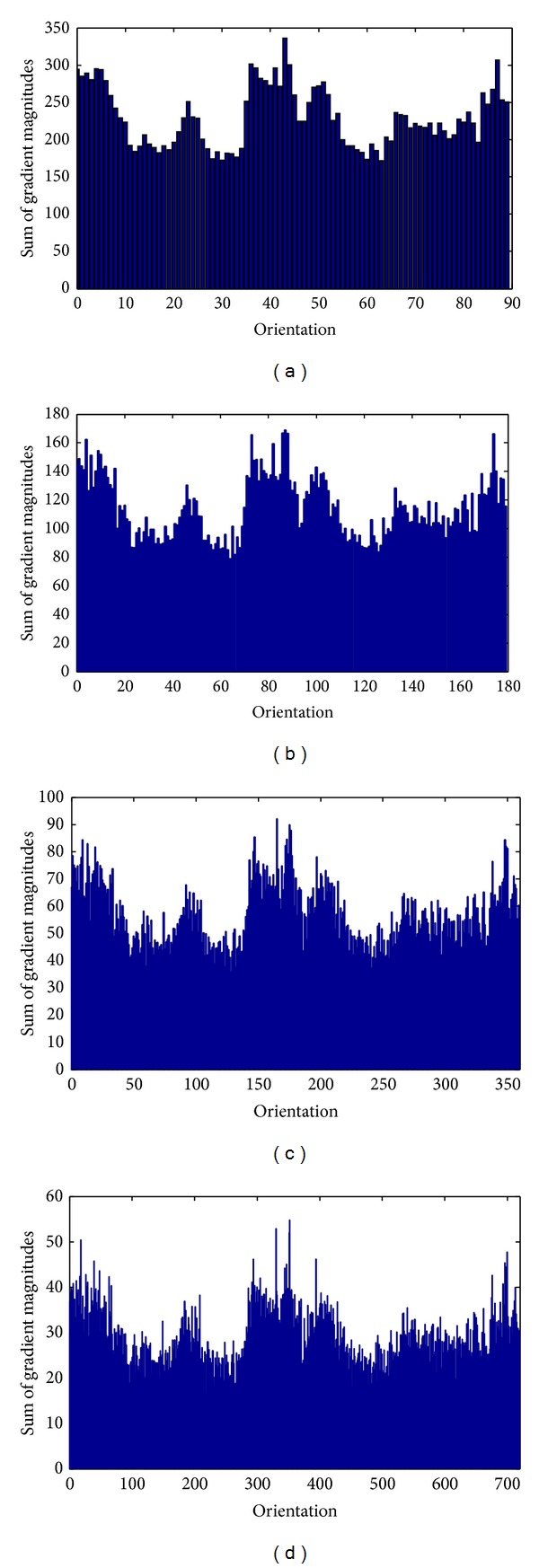
Magnitudes weighted orientation histograms with different orientation bins. (a) *K* = 90, (b) *K* = 180, (c) *K* = 360, and (d) *K* = 720.

In Figure 5, a test was shown to generate magnitudes weighted orientation histograms with different orientation bins for brain T2 MR as shown in Figure 2. Let *K* denote the number of bins for the orientation histogram. The orientation bins *K* were selected from 90, 180, and 360 to 720. Correspondingly, the orientation resolutions of the histograms were 4°, 2°, 1°, and 0.5° (360/*K*). To clearly observe the accumulation of histograms in different orientation bins, the type of bar was adopted for histogram presentation rather than using the conventional type of line. Magnitudes weighted orientation histograms with different orientation bins were created in Figure 5.

### 2.3. Histogram Matching

Since two images may not cover exactly the same parts of anatomic structures, in order to maximize the area of overlap between the two images, areas within largest circular regions centered at the images are used in the orientation histogram accumulation. To maximize the area of overlap between the images, only areas within largest circular regions centered at the images are used for the accumulation. The orientation histogram of one image is cyclically slided over the other orientation histogram from the other image, and the position where the two histograms best match is used to determine the rotational difference between the images, assuming *H*_*A*_ and *H*_*B*_ are the magnitudes weighted orientation histograms of the reference image and the moving images, respectively. Histogram matching is to determine the optimal translation that minimizes the cost function *T*_*D*_ of two histograms *H*_*A*_ and *H*_*B*_, that is, to find *T*_*D*_(*j*) = ∑_*i*=0_^*K*−1^(|*H*_*A*_(*i*) − *H*_*B*_(*i* + *j*)|) for *j* = 0,…, *K* − 1. Then find *J* such that *T*_*D*_(*J*) = min⁡⁡(*T*_*D*_(*j*)) for *j* = 0,…, *K* − 1, where *J* is the estimation of rotational difference between two images and *K* is the number of bins for the orientation histogram. Note that this shift of histogram matching is done cyclically, so that if *i* + *j* > *K* − 1, then *i* + *j* is replaced with *i* + *j* − (*K* − 1). The matching metric is L1 norm of *H*_*A*_ and *H*_*B*_. In addition, L2 norm is also performed for the histogram matching *T*_*D*_(*j*) = ∑_*i*=0_^*K*−1^(|*H*_*A*_(*i*)−*H*_*B*_(*i*+*j*)|^2^).

**Figure 6:**
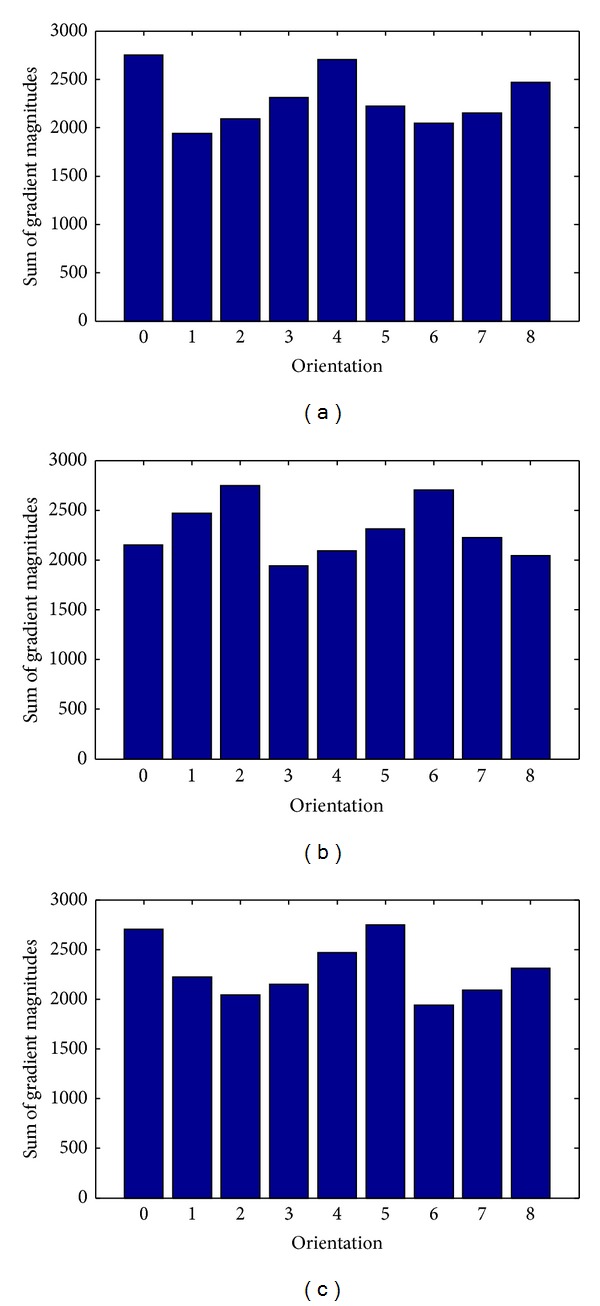
Cyclically histogram shift by Fourier shift theorem. (a) Original histogram. (b) Cyclically shift *j* = 2. (c) Cyclically shift *j* = 5.

Fourier transform based on Fourier shift theorem was used to efficiently compute the sum of absolute difference (L1 norm) or the sum of squared difference (L2 norm) for all possible shifts cyclically for *j* = 0,…, *K* − 1.  Figure 6 showed the process of cyclically shift of histograms by Fourier shift theorem in the implementation. To better visualize the cyclically shift of the histogram, orientation bins *K* was chosen as 9 to generate the magnitudes weighted orientation histogram for orientation bins for brain T2 MR. The shift *j* was considered as 2 and 5, respectively. The histogram matching values of *T*_*D*_ with shift *j* from 0 to *K* − 1 were recorded, and the global minimum value of *T*_*D*_ was considered as the position of the best match. In the implementation, a figure of *T*_*D*_ with all shifts was plotted for better observation, where the horizontal axis was the shift *j* from 0 to *K* − 1, and the vertical axis was the histogram matching value *T*_*D*_. In order to improve the robustness of histogram matching and eliminate the noise in orientation histograms, 1-dimensional Gaussian smoothing with small standard deviation was performed for two histograms before the matching process. Finally, interpolation methods were used to improve the accuracy of orientation difference estimation. The point which corresponded to the global minimum value and its nearest neighborhood points in the histogram of *T*_*D*_ were fitted as a curve to generate the minimum in the continuous domain.

**Figure 7:**
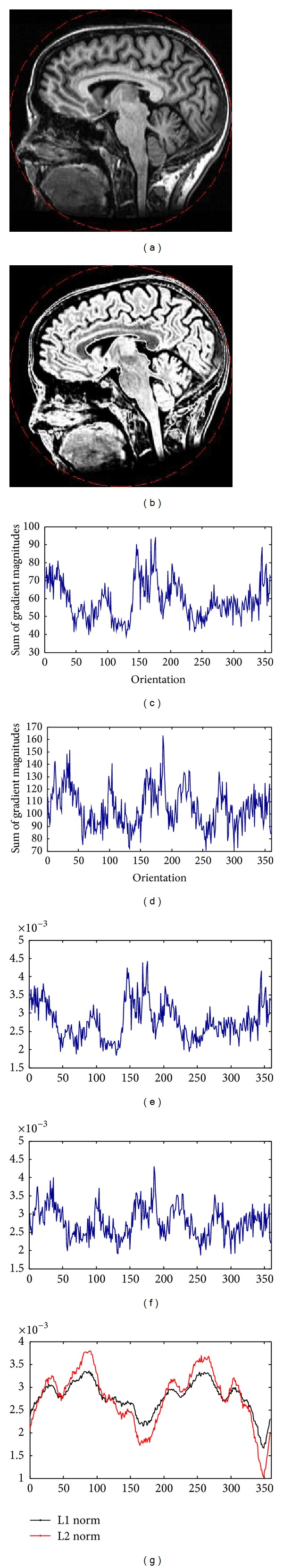
Simulated orientation test by gradient magnitude weighted orientation histogram matching of T1 MRI (Figure 2(a)) and rotated T2 MRI (Figure 2(c)) images. (a) and (b) are the original images and the red circle which is largest centered at the images shows the area of overlap for orientation histogram accumulation. (c) and (d) are magnitude weighted orientation histograms for (a) and (b), respectively. (e) and (f) are the normalized histograms of (c) and (d), respectively. (g) shows the values of cost functions T of histogram (e) and (f) with shift *j* for *j* = 0,…, *K* − 1. The similarity metric is tested by L1 and L2 norm, respectively. The minimum value of the cost function T corresponds to *J* = 349 along the horizontal coordinate. The obtained orientation difference is 11°, which is very close to its desired value.

A synthetic example of determining the rotational difference between two brain T1 MRI and T2 MRI images was presented to demonstrate the detailed performance of the proposed algorithm in Figure 7. The synthetic rotational difference between the two images Figures 2(a) and 2(c) was 11.46°. The detailed results of generated histograms and matching were shown in Figure 7, and the comparison of L1 norm and L2 norm for histogram matching was also shown. From the test results, the obtained result generated by the proposed method was 11°, which was very close to the desired value. In addition, both L1 norm and L2 norm achieved similar performances.

## 3. Experiments

To demonstrate the accuracy and robustness of the proposed method for global orientation difference estimation between multimodality medical images, both synthetic and clinical medical images were applied for the experiments. The performance of the proposed method on synthetic images was firstly tested as the ground truths of the rotational difference were known. The gold-standard rigid-body rotational difference for each registration was set by rotating one image in the image pair with respect to the other image. The proposed method was compared with the edge-based method for rotational estimation for challenging multimodality images. Finally, we incorporated the proposed orientation estimation for preregistration of multimodality images.

### 3.1. Synthetic Test

**Figure 8:**
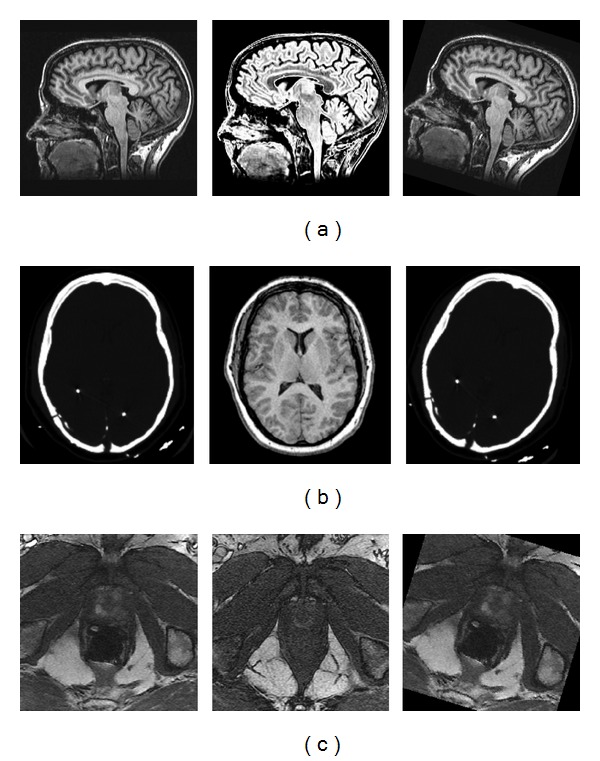
Image pairs used to evaluate the registration method. The first column and the second column were the original image pairs and the third column was generated by rotating the first column image with fixed 17° clockwise. (a) Brain T1/T2 MRI with intensity and slight scale difference. (b) Head CT/MR with significant differences. (c) Prostate MR with deformations and partial occlusions due to the surgery.

The method was tested in three different medical image processing scenarios: brain T1WI and T2WI with scale difference (Figure 8(a)), head CT image to the T1 weighted MRI image in the “head” image pair (Figure 8(b)), and MR prostrate image pair with large local and global deformation (Figure 8(c)). It was assumed that the ground truths of rotational differences between images were acquired by computer generation, so the first image was rotated by a fixed degree using the bicubic interpolation to generate the third image. Hence, firstly the rotational difference between the original image pairs was tested and then the result between the generated image and the second image was tested as well. Without loss of generality, the fixed degree of 17° clockwise was set for all cases and the generated third image was shown in each case.

**Figure 9:**
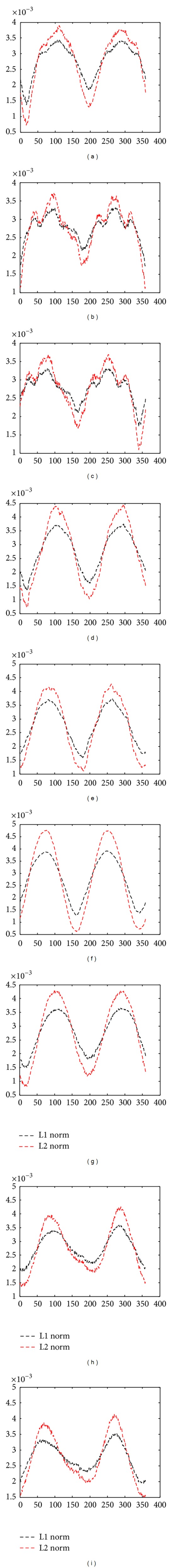
Cross correlation of gradient magnitudes weighted orientation histogram for each image pair.

The test results of rotational differences between image pairs shown in Figure 8 were reported in Table 1. The cross correlation of gradient magnitudes weighted orientation histograms for each image pair were also presented in Figure 9 correspondingly. It was known that the third column was generated by rotating the first column image with fixed 17° clockwise; the test results of the first column image and third column image were also shown to demonstrate the accuracy of the proposed method for monomodality images. Note that the rotational differences between the original image pairs (1st column and 2nd column) were slightly small from the observation; the test results of orientation difference estimation as shown in the Table 1 were consistent to the observation, which were very close to the desired value. The proposed method of rotational estimation was applicable to multimodality images in Figures 9(a) and 9(b). Since the proposed method did not require any feature extraction or segmentation for preprocessing to acquire the rotational difference between images, it was efficient and simple for preregistration of both monomodality and multimodality images. From the results of images in Figure 8(c), the proposed method was robust to considerable global and local deformations in medical images, which frequently appeared in biomedical image applications.

**Figure 10:**
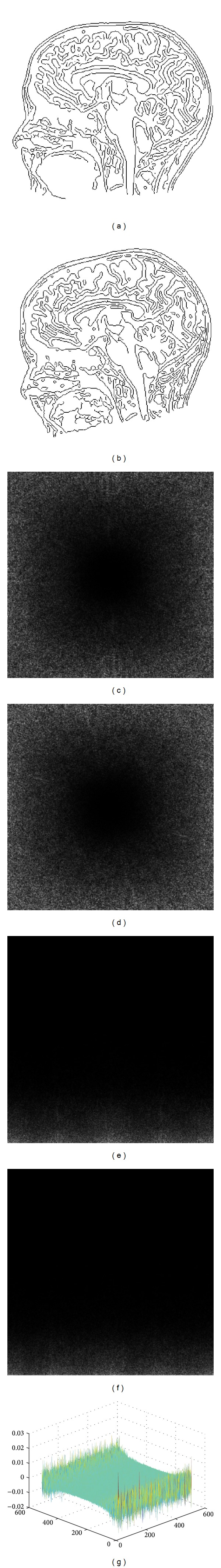
Simulated orientation test by edge-map based method of T1 MRI (Figure 2(a)) and rotated T2 MRI (Figure 2(c)) images. (a) Canny edge map of Figure 2(a). (b) Canny edge-map of Figure 2(c). (c) Magnitude spectrum of edge map of (a) in frequency domain. (d) Magnitude spectrum of edge map of (b) in frequency domain. (e) Log-polar resample of (c). (f) Log-polar resample of (d). (g) Phase-correlation between (e) and (f). Note that the peak value corresponded to the rotational angle 11.25°, which was also very close to the desired value.

**Figure 11:**
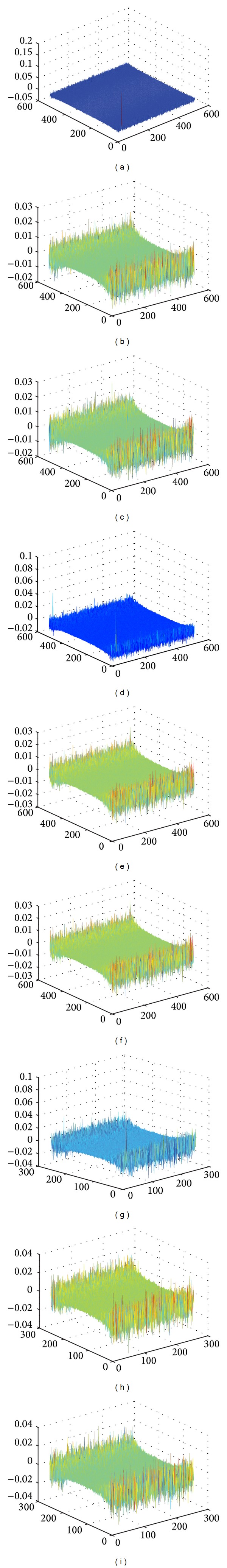
Phase correlation of the polar presentation of Fourier spectrum for each image pair.

Then, test results of image pairs in Figure 10 by the edge-map based method [15] were tabulated in Table 2. In the implementation of the edge-map based method, note that phase correlation [26] was used to determine the rotation between images rather than using the cross correlation recommended in the original paper due to the fact that phase correlation can generate the same results as cross correlation but is more robust than cross correlation with respect to partial occlusion [27]. As shown in Figure 11, the phase correlation of the polar presentation of Fourier spectrum for each image pair was displayed correspondingly. Since the images of the 3rd column were generated by rotating the images of the 1st column through the bicubic interpolation, the edge-maps were very similar for these two image sets. Therefore, the rotational differences between such two image sets detected by the edge-map based method were very accurate as shown in the Table 2. Specifically, their phase correlation of the polar presentation of Fourier spectrums had distinctive peaks in Figures 11(a), 11(d), and 11(g), which corresponded to correct estimation of rotational differences. However, the results of other cases were all incorrect. For the brain T1/T2 MRI images, there was no distinctive peak in the field of phase correlation due to the fact that there were slight scale differences between two images. For the CT and MR “head” image, their edge-maps were significantly different, and therefore the detected results were incorrect due to the lack of abundant similar edges. For the Prostate MR images, the results were also incorrect due to the considerable global and local deformations between images. Therefore, it was experimentally verified that the edge-map based method was extremely limited to acquire the rotational difference between images. Comparatively, the proposed method was based on the global similarity of local information in spatial domain, which was very robust to multimodality images with respect to nonlinear deformation, slight scale differences, and considerable dissimilarities.

In essence, the proposed method of rotational estimation is a general technique for image registration with wide ranges of rotational differences. In order to demonstrate the sensitivity of the method to smaller rotations and larger rotations, detailed simulation tests were presented with a smaller range of rotations, including 1°, 2°, 3°,…, 8°. Meanwhile, larger rotations were also tested, including 40° or 70°. Three medical images were selected for the test including brain T1 MRI, head CT, and prostate MR images as shown in Figure 8. Rotating one test image with a synthetic rotational angle by the bicubic interpolation would generate its rotated image for the rotational estimation. The experimental results were reported in Table 3. Note that the standard deviation of Gaussian derivative *σ* should be relatively large (e.g., *σ* = 5.0 in the test) in order to reduce the influence of interpolation artifacts from the bicubic interpolation. From the test results, the proposed method obtained promising results when the rotational difference was larger than 3°. However, the proposed method was not much sensitive to too small rotational differences, for example, <2°. When the rotational difference between two images was smaller than 2°, the obtained result was usually zero. Two reasons accounted for it. One was that the orientation resolution of histograms is 1° in the work, and it was so difficult to differentiate too small rotational differences from the orientation histogram. Another factor was that the precision was strongly related to the interpolation method, which in the case was bicubic interpolation. It was also very difficult to generate rotational images precisely with very small known rotational differences (e.g., 1 or 2°) by the bicubic interpolation. Therefore, when the rotational difference was very small (e.g., <3°), the test result may contain small errors (e.g., error < 2° experimentally). But these results with such small rotation errors were mostly accurate to guide the iterative optimization to obtain desired values, because the estimated value was very close to the desired value.

### 3.2. Application in Clinical Image Registration

The proposed orientation detection was experimentally incorporated into the registration of CT/MR and T1/T2 MRI images. Four clinical cases with multimodality images under affine transformation were registered. The image registration process was performed on a computer with CPU 3.3 GHz and 4G RAM. The computation time of the registration process with and without the proposed method was measured, and the time consuming of the proposed technique was also reported in Table 4. It was found that the consuming time of optimization iteration was reduced significantly due to the incorporation of the proposed orientation detection. The consumed time of the proposed method was much less than the time consumed by iteration optimization. Since the initial value of orientation difference is accurately estimated by the proposed method, the initial value for the iterative optimization becomes closer to the desired value. Therefore, fewer times of iterations were required to approach the global minimum for the iterative optimization. Therefore, the utilization of the proposed scheme dramatically decreased the time for iteration and speeded up the registration process with the proposed orientation detection as shown in Table 4.

**Figure 12:**
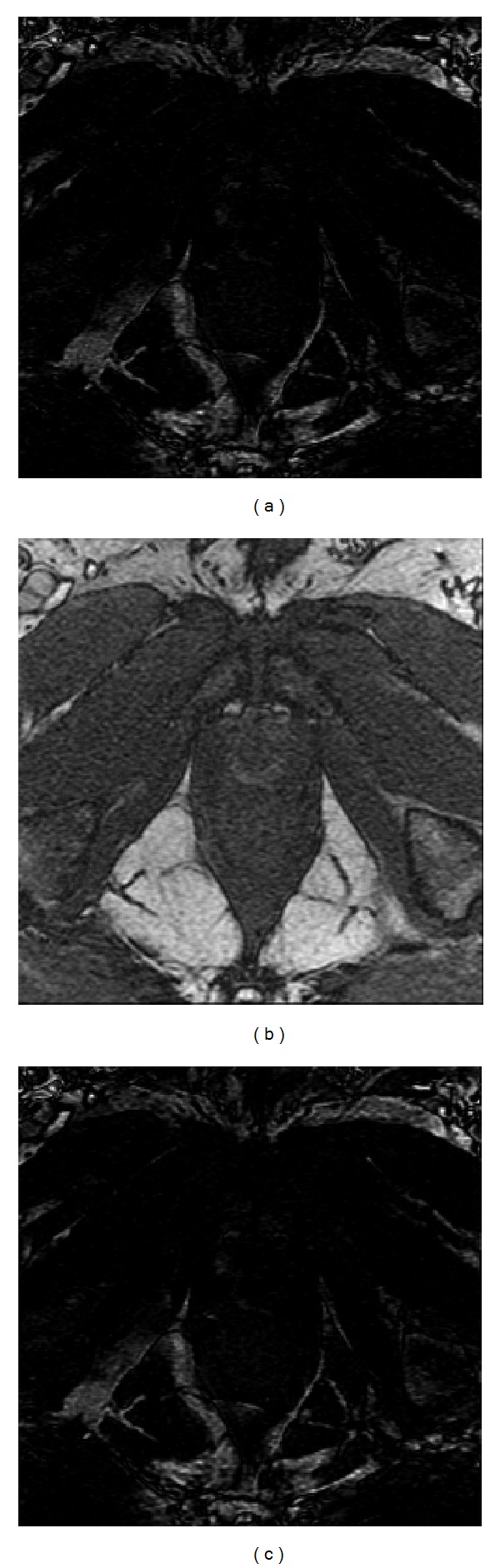
Experimental results of prostate image registration under affine transformation without prior information of orientation difference. (a) Difference of sense image and reference image, SSD = 0.0314. (b) Transformed image. (c) Difference of transformed image and reference image, SSD = 0.0302. Iteration times of optimization: 12 iterations.

**Figure 13:**
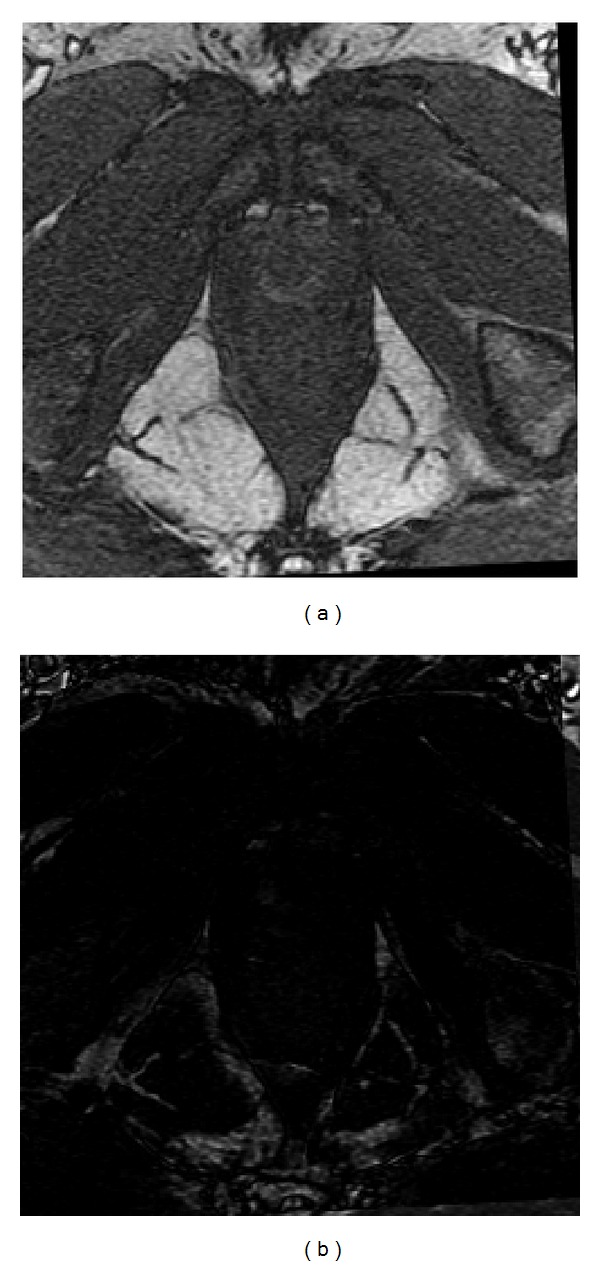
Experimental results of prostate image registration under affine transformation with the proposed orientation detection. (a) Transformed image. (b) Difference of transformed image and reference image, SSD = 0.0269. Iteration times of optimization: 120 iterations.

An image pair is often aligned initially based on a rigid or affine transformation before a nonrigid transformation for deformable medical image registration. Therefore, registration results of clinical prostate images as shown in Figure 8(c) were also shown to demonstrate the performance of the proposed method for preregistration to deformable image registration. In Figures 12 and 13, the preregistration using affine transformation with the similarity metric of squared sum of difference (SSD) [8] was performed for the original prostrate images. The absolute difference image between the sensed image and the reference image before the registration was calculated as shown in Figure 12(a), and also the difference image between the transformed image and the reference images after the registration was calculated. Regions with higher intensities in the difference image showed larger difference between the two images in corresponding regions, and a successful registration should decrease intensity values of overlap areas in difference images. It could be observed that the difference image between the transformed image and the reference image as shown in Figure 12(c) did not have much changes compared with the difference image between the sensed image and the reference image as shown in Figure 12(a). The evaluation metric SSD of registration performance before and after the registration also had very little changes due to the failed optimization. The results from the optimization cannot converge to the global minimum without any prior information of orientation, and the iteration was stopped quickly because the initial value was not near the desired value and easily fell into the local minimum.

Figure 13 showed the registration results for the prostate images with the orientation estimation by the proposed method. The registration performance became much better than before, since the SSD becomes relatively smaller. Meanwhile, more regions in the difference image between the transformed image and the reference image as shown in Figure 13(b) became black, and it also indicated that the iteration of the optimization process attained the global minimum due to the fact that the initial value was close to the desired value with the known orientation difference in advance. Experimental results from Figures 12 and 13 showed that the proposed method could significantly improve the chances of finding the global minimum of the registration. Therefore, the proposed technique is directly applicable to preregistration before deformation image registration and makes the registration process more robust and reliable.

Since the goal of preregistration by the proposed method is to reduce the search space of the iteration optimization and increase the robustness of the process of the registration. The purpose of using SSD in Figures 12 and 13 was to verify that the proposed method could significantly improve the chances of finding the global minimum of the registration. In Figure 12, the iterative optimization stopped at a local minimum with 12 iterations because the initial value is far away from the desired value. Conversely, the iterative optimization can find the global minimum of registration with the aid of the proposed method for initial estimation. The registration results of the global minimum (SSD 0.0269) appeared to be better than the local minimum (SSD 0.0302) from the experiments. Therefore, experimental results demonstrated that the proposed method could be incorporated into the general image registration and improve the chances of finding the global minimum of the registration (robustness).

### 3.3. Discussion

It is striking that the estimation of rotational differences is accurate and robust for very dissimilar images captured for the same scene. When there are large local differences, illumination changes, scale or rotational changes, the process of histogram matching generates promising results. The main reason is that the proposed method is computed based on the global distribution of local orientations weighted by gradient magnitudes, which seems to be robust to considerable image changes for multimodalities. The statistic orientation histogram matching is robust to capture the rotational differences between images. Since no high-level features in images are required in the process, the proposed method is very simple and robust to determine the rotational difference between two images. The proposed method for orientation differences estimation is able to be used for rigid registration directly. Since if the orientation difference between two images is known, the prealignment becomes significantly easier after eliminating the rotational difference between two images. The work left from translation estimation is then determined by mutual information-based [28], phase correlation [26, 27] based, or cross correlation [29] based template matching. The proposed method can also be used in the preregistration for deformation medical image registration due to the fact that the robustness and reliability of the method make it possible to register very challenging and dissimilar images because the orientation between images could be obtained in advance even for very dissimilar images in multimodalities. The incorporation of the prior information of orientation detection into general medical image registration [30] also reduces the search space of optimization and enhances the robustness of the registration calculation.

**Figure 14:**
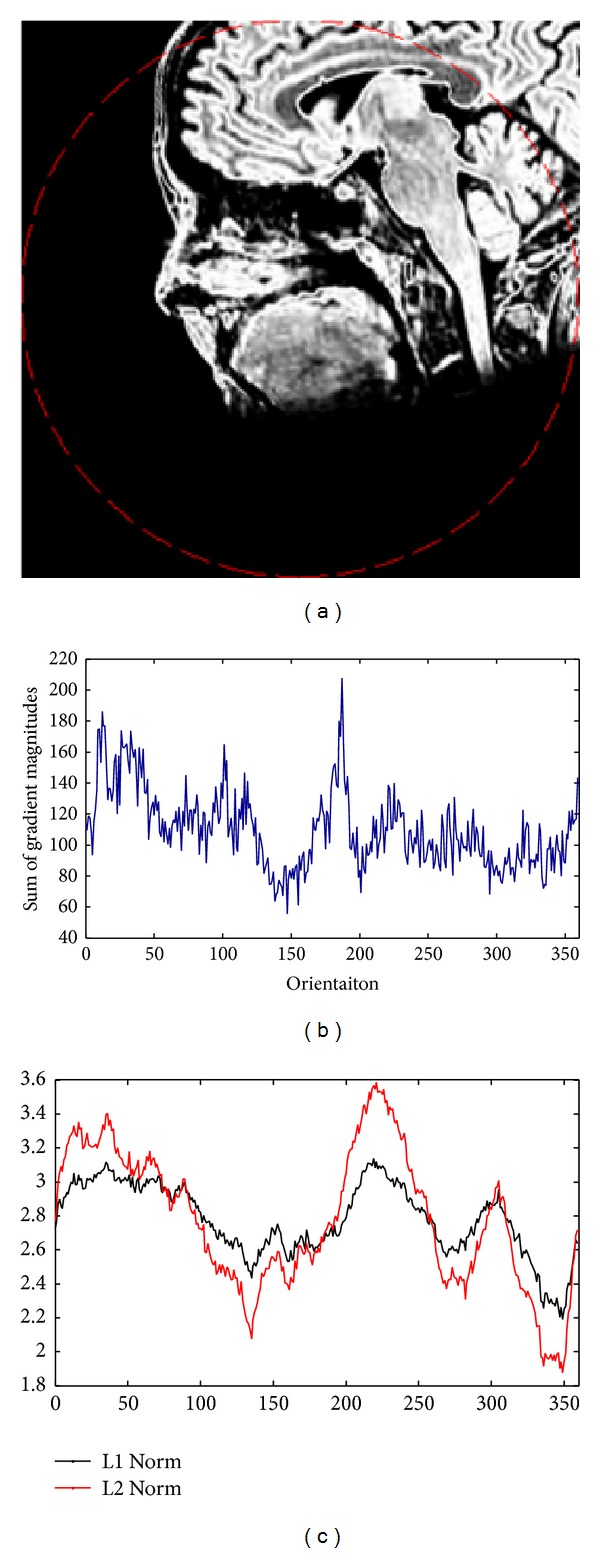
Simulated test result of partial occlusion. (a) Shifted T2 MRI image which was generated by synthetically shifting the content of rotated T2 MRI. (b) Magnitude weighted orientation histogram of shifted T2 MRI image. (c) shows the values of histogram matching. The similarity metric is tested by L1 and L2 norm, respectively. The minimum value of the cost function T corresponds to *J* = 349 along the horizontal coordinate. The obtained orientation difference is 11°.

The overlapping area between two unregistered images is an important factor for general image registration. It is often assumed that the overlapping area is larger than 50% of the image area, and registration methods have to be robust to partial occlusion. In clinical medical images, the scan volumes to be registered often contain same anatomic structures of the patient but are often scanned at different time or in different scans. Therefore, it is assumed that the unregistered two images are almost contain the same scene in medical images, so the red circle in Figure 7 is set to be the largest circular region of the image as the overlapping area for accumulation in the proposed method. A further test of partial occlusion for the proposed method was shown in Figure 14. Two brain T1 MRI and rotated T2 MRI images in Figures 7(a) and 7(b) were used for the test, but the content of rotated T2 MRI was synthetically translated to be the shifted T2 MRI image as shown in Figure 14(a). Red circle showed the largest circular region for accumulation. Nonoverlap area between T1 MRI image and shifted T2 MRI image was relatively large.  Figure 14(c) showed the values of cost functions T of histogram correlation with shift *j* for *j* = 0,…, *K* − 1. The global minimum value was 349 (or 11°), which was same to the result in Figure 7. The experiment result demonstrated that the proposed method was very robust to partial occlusion.

**Figure 15:**
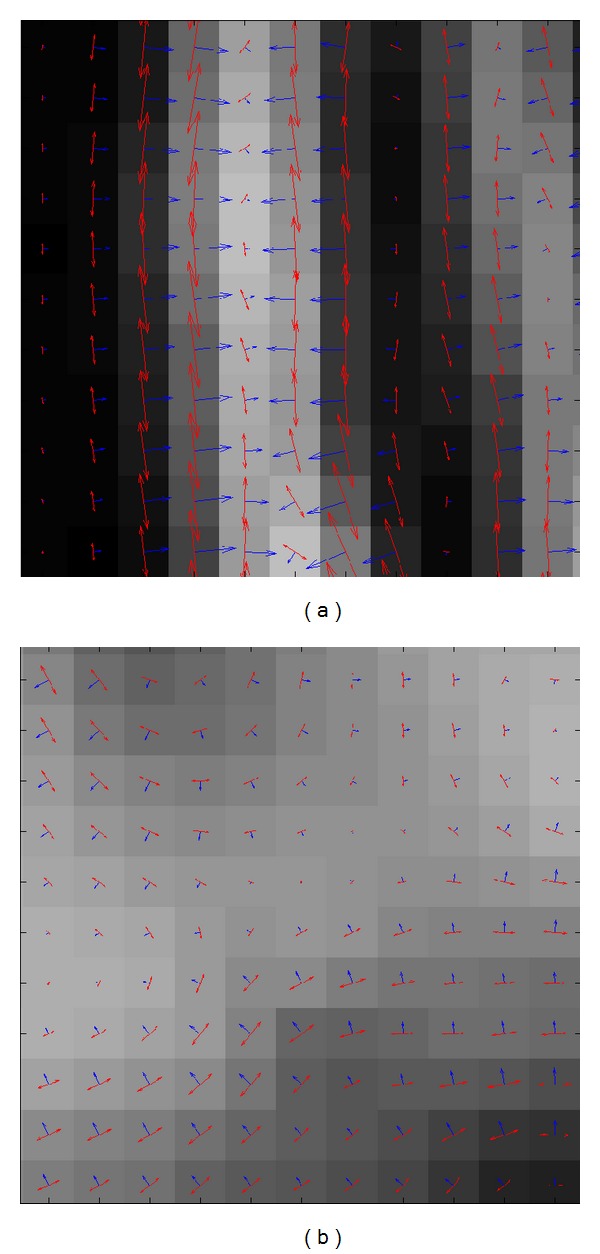
Local orientations of patterns in T1 MRI (Figure 2(a)). (a) Straight pattern. (b) Curved pattern. Note that the blue arrow shows the local gradient orientation at each pixel, and the length of the blue arrow denotes the value of local gradient magnitude.

Since the gradient orientations in a neighborhood of a straight pattern have (on average) opposite directions, the orientation histogram appears to be symmetric from 0 to 360° due to the fact that about half the gradient vectors have an opposite direction (180°) with respect to other halves for straight patterns in images.  Figure 15(a) showed the local orientations of straight patterns in T1 MRI image, and pixels on one side of the straight line have totally opposite directions with respect to the pixels on the other side. Generally, medical images contain complex patterns including both straight patterns and curved patterns. Therefore, the accumulated orientation histogram is usually asymmetric since the curved patterns do not have an opposite direction as depicted in Figure 15(b), and the global minimum of histogram matching corresponds to the desired value experimentally. When the orientation histogram is symmetric if there are mainly straight patterns in images, there will be several local minimums in the process of histogram matching. To make sure that there is no systematic error for symmetric histogram matching, both the global minimum *θ* and *θ* + 180° should be considered for image registration. Since the rotational angle for clinical medical images is often not too large, the global minimum *θ* can be validated by this experience.

The major difference in the image registration with and without the proposed method is the computation iterations (efficiency) as well as the possibility of finding the global minimum of the optimization (robustness), rather than the accuracy. Thus, reducing the number of iterations of optimization (time consuming) is one advantage of the proposed method. More importantly, increasing the possibility of finding global minimum of optimization of image registration (robustness) is another advantage of the proposed method. Certainly, accuracy of registration is known to be most important in image registration, but the robustness and efficiency of registration are very important as well. Modern computers with more powerful performances may make the process of time reducing meaningless. However, the success rate of the registration (robustness) cannot be improved with powerful computers. Experiments have demonstrated that the proposed method can make the registration more reliable in the process of iterative optimization and reduced the chances of registration failure.

This application is expected to straightforwardly extend to 3D volumetric images, in which the local orientation is estimated based on the smoothed structure tensor [31] in a voxel wise manner, followed by separating a vector of the local orientation *V* in 3D into three components *V* = {*θ*, Ψ, *γ*}, where *θ*, Ψ, and *γ* are the angles between the vector and the *x*, *y*, and *z* coordinate, respectively. The orientation difference in 3D is thus converted to the histogram matchings with respect to *θ*, Ψ, and *γ*, from which the final orientation difference between two 3D volumetric images is defined by synthesizing Δ*θ*, ΔΨ and Δ*γ* in the *x*-*y*-*z* coordinate system. Without loss of generality, the case of 2D image registration has been fully considered for explanation in the work.

## 4. Conclusion

In this work, a very simple and robust method was proposed to compute the rotational difference between two multimodality images and very dissimilar monomodality images. The proposed method is superior to the existed edge-map based method from the experimental comparison. Experimental results have demonstrated that orientation detection between images is reliable and robust to considerable deformations, slightly scale differences, and dissimilar image contents. Experimental results have also demonstrated that the incorporation of this method into image registration both enhances the robustness of registration and significantly speeds up the registration calculation. It is worthwhile to note that the proposed method of orientation detection is appropriate to be applied for image registration with rigid transformation and nonrigid transformation, which has very broad applications in medical image registration and other applications in general image registration.

## Figures and Tables

**Table 1:** Test results of rotational differences between image pairs by the proposed method.

Image pair	1st column and 3rd column *γ* (°)	1st column and 2nd column *α* (°)	2nd column and 3rd column *β* (°)	Absolute value |α − β|
Brain T1/T2 MRI	17 Figure 9(a)	358 (or −2) Figure 9(b)	340 Figure 9(c)	18
Head CT/MR	17 Figure 9(d)	357 (or −3) Figure 9(e)	341 (or −19) Figure 9(f)	16
Prostate MR	17 Figure 9(g)	7 Figure 9(h)	353 (or −7) Figure 9(i)	14

**Table 2:** Test results of rotational differences between image pairs by edge-map based method.

Image pair	1st column and 3rd column *γ* (°)	1st column and 2nd column *α* (°)	2nd column and 3rd column *β* (°)	Absolute value |α − β|
Brain T1/T2 MRI	16.87 Figure 11(a)	120.23 Figure 11(b)	82.97 Figure 11(c)	27.26
Head CT/MR	16.87 Figure 11(d)	355.08 Figure 11(e)	46.40 Figure 11(f)	308.68
Prostate MR	16.87 Figure 11(g)	68.90 Figure 11(h)	22.50 Figure 11(i)	46.40

**Table 3:** Rotational estimation results of image pairs with synthetic rotational angles.

Synthetic angles	Test image		
	Brain T1	Head CT	Prostate MR
1°	0°	0°	0°
2°	1°	2°	1°
3°	3°	4°	3°
4°	4°	4°	4°
5°	5°	5°	5°
6°	6°	6°	6°
7°	7°	7°	7°
8°	8°	8°	8°
40°	40°	40°	40°
70°	70°	70°	70°

**Table 4:** Comparison of the iteration time of optimization with and without the proposed method.

	Iteration times of optimization without the proposed method	Iteration times of optimization with the proposed method	Consumed time of the proposed method (second)
Case 1	46 times (15.69 seconds)	23 times (8.08 seconds)	0.504 seconds
Case 2	109 times (36.54 seconds)	26 times (8.31 seconds)	0.522 seconds
Case 3	226 times (75.56 seconds)	25 times (8.64 seconds)	0.566 seconds
Case 4	44 times (15.17 seconds)	24 times (8.23 seconds)	0.506 seconds
